# Identity as a psychological compass: the mediating role of resilience in international students’ college adjustment amidst culture shock

**DOI:** 10.3389/fpsyg.2025.1668553

**Published:** 2025-09-16

**Authors:** Weimin Yang, Lili Gao

**Affiliations:** ^1^Jiangsu Vocational Institute of Commerce, Nanjing, China; ^2^Business School, Hohai University, Nanjing, China

**Keywords:** international students, culture shock, college adjustment, identity, psychological resilience

## Abstract

**Introduction:**

The rapid increase in international students in China underscores the imperative to understand their successful adaptation. This study investigates the roles of identity and psychological resilience in facilitating college adjustment among international students confronting culture shock in China.

**Methods:**

Drawing upon Social Identity Theory and Identity Consolidation Theory, we hypothesized that a strong sense of identity would positively influence psychological resilience, mediating the relationship between identity and college adjustment. A quantitative survey was conducted with 2,097 international students in various universities across China.

**Results:**

The findings indicate that identity significantly predicted higher levels of psychological resilience, and psychological resilience, in turn, was a strong predictor of better college adjustment. Furthermore, psychological resilience significantly mediates the relationship between identity and college adjustment, suggesting that a well-defined identity contributes to resilience, equipping students to navigate the challenges of culture shock and adapt successfully to university life.

**Discussion:**

These results provide valuable insights for developing targeted support programs and interventions to enhance international students’ well-being and successful integration in China.

## Introduction

China has emerged as a prominent global destination for international students, with its rapidly developing economy, world-class universities, and increasing international influence attracting a diverse population of scholars from across the globe. While this influx enriches Chinese universities’ academic and cultural landscape, international students often face unique challenges inherent in cross-cultural transitions. A primary among these is culture shock, a disorienting experience characterized by anxiety, frustration, confusion, and feelings of alienation resulting from exposure to an unfamiliar way of life, language, social norms, and academic systems (Bai and Wang, 2024; Presbitero, 2016). Navigating this complex psychological and social landscape is crucial for their successful college adjustment, encompassing academic, social, personal-emotional, and cultural adaptation (Polat and Arslan, 2022).

Unsuccessful adjustment can lead to various adverse outcomes, including academic difficulties, psychological distress (e.g., anxiety, depression), social isolation, and even early withdrawal from studies (Sharp and Theiler, 2018). These challenges have been amplified in the post-pandemic era, with studies highlighting increased feelings of isolation and uncertainty among international students, thus underscoring the heightened need for psychological resources like resilience (Bozkurt et al., 2022). Therefore, understanding the protective factors that facilitate international students’ adaptation in the face of culture shock is a critical area of research. This study focuses on two such pivotal factors: identity and psychological resilience. It explores their interplay in predicting college adjustment among international students in China.

As a multifaceted construct, identity refers to an individual’s sense of self, encompassing personal characteristics, social roles, and group affiliations (Tajfel and Turner, 2004). For international students, identity can be particularly complex. This study conceptualizes identity through two key dimensions: a social dimension, operationalized as school identity (i.e., a sense of belonging to the university community), and a personal dimension, operationalized as self-identity (i.e., a coherent understanding of one’s values, beliefs, and goals). This dual focus allows for examining how both group affiliation and a stable sense of self contribute to adaptation (Zhang and Ting, 2025). Drawing on Social Identity Theory (Tajfel and Turner, 2004), a strong sense of belonging to a positive social group (e.g., fellow international students, a specific national group within China, or even the broader university community) can provide social support, boost self-esteem, and offer a framework for understanding and navigating the new environment, thereby mitigating the effects of culture shock. Furthermore, Identity Consolidation Theory (Schwartz, 2007) suggests that a coherent and integrated sense of self, resulting from successful exploration and commitment to various identity domains, provides a stable psychological foundation. For international students, this might involve reconciling aspects of their home culture with new experiences in China, leading to a more resilient and adaptable self. A well-consolidated identity can offer a sense of purpose and direction, crucial for coping with the uncertainties of a new cultural context.

Psychological resilience is the ability to adapt well in the face of adversity, trauma, tragedy, threats, or significant sources of stress (Vella and Pai, 2019). For international students, resilience manifests as the capacity to “bounce back” from the stresses of culture shock, maintain well-being, and progress academically and personally. Research consistently shows that resilient individuals are better equipped to cope with stress, solve problems, and utilize coping resources effectively (Connor and Davidson, 2003). It is theorized that a strong and coherent identity may foster psychological resilience by providing a stable internal compass, a sense of self-worth, and a framework for interpreting challenging experiences constructively. This enhanced resilience then serves as a crucial buffer against the negative impacts of culture shock, enabling more successful college adjustment (Rajan-Rankin, 2014).

While existing literature has explored aspects of international student adjustment, identity, and resilience independently, the specific mediating role of psychological resilience in the relationship between identity and college adjustment, particularly within the unique context of international students navigating culture shock in China, remains underexplored. Previous studies often focus on a single aspect or do not fully capture the dynamic interplay of these factors. This study aims to fill this gap by proposing a model where identity directly influences psychological resilience, and this enhanced resilience, in turn, directly contributes to better college adjustment.

Therefore, the primary objectives of this study are: (1) To examine the direct relationship between identity and psychological resilience among international students in China. (2) To investigate the direct relationship between psychological resilience and college adjustment among international students in China. (3) To explore the mediating role of psychological resilience in the relationship between identity and college adjustment for international students confronting culture shock in China.

This research employs a quantitative approach, utilizing survey data from a large sample (*N* = 2,097) of international students studying in China. The findings are expected to contribute significantly to cross-cultural psychology, international education, and student development. Theoretically, it will deepen our understanding of identity formation and resilience in multicultural contexts. Practically, the insights gained will be invaluable for universities, student support services, and policymakers in China to design and implement more effective interventions and support programs tailored to the specific needs of international students, thereby fostering their successful integration and overall well-being. The subsequent sections of this paper will detail the methodology, present the results, discuss the findings, and outline the study’s limitations and implications for future research.

## Literature review and hypotheses development

### College adjustment of international students

College adjustment is a multifaceted and dynamic process fundamental to students’ academic success, personal development, and overall well-being throughout their university journey. It encompasses a student’s capacity to effectively adapt to the new academic, social, and personal-emotional demands inherent in a higher education environment (Baker and Siryk, 1989; Banyard and Cantor, 2004). While all students navigate this complex transition, the process is significantly more intricate for international students, involving an additional, critical layer of cultural adaptation that profoundly shapes their overall experience (Polat and Arslan, 2022).

Traditionally, research on college adjustment identifies several key dimensions. Academic adjustment refers to students’ ability to meet the intellectual demands of their studies, encompassing aspects such as academic performance, effective study habits, engagement with coursework, understanding of pedagogical expectations, and successful interactions with faculty (Clinciu and Cazan, 2014). Challenges in this area often stem from differing teaching styles, language barriers, or unfamiliarity with the host country’s educational system. Concurrently, social adjustment pertains to a student’s comfort and success in forming relationships and integrating into the social fabric of the university community. This involves developing a sense of belonging, establishing peer support networks, participating in campus activities, and navigating new social norms (Schwitzer et al., 1999). Difficulties in social integration can lead to feelings of loneliness, isolation, and a diminished sense of community. Beyond academic and social spheres, personal-emotional adjustment is crucial, encompassing a student’s psychological well-being and capacity to manage university life’s emotional and psychological demands. This dimension includes coping with stress, maintaining emotional stability, developing self-efficacy, managing newfound independence, and addressing issues such as homesickness, anxiety, or depression (Estrada et al., 2006). Success in this area is paramount for maintaining overall mental health and fostering resilience against life’s challenges.

For international students, navigating a new cultural environment that often differs significantly from their home country introduces a profound additional dimension: cultural adjustment. This involves a continuous process of understanding and adapting to local customs, social norms, communication styles (both verbal and non-verbal), daily routines, and cultural values that may be vastly different from their own (Polat and Arslan, 2022). Language barriers, even for those with prior proficiency, can create subtle yet significant hurdles in academic comprehension and social interaction, impacting their ability to integrate fully. Furthermore, the greater the perceived “cultural distance” between native and host cultures, the more challenging the adjustment process can be, often leading to confusion, frustration, and alienation, commonly known as culture shock (Jin and Acharya, 2022). International students also frequently engage in identity negotiation, grappling with their personal and cultural identities as they immerse themselves in a new environment, which can lead to a re-evaluation, strengthening, or even a transformation toward a bicultural identity (de la Serna, 2022).

### School identity and college adjustment

School identity, also called institutional identity or school belongingness, refers to the degree to which individuals identify with and feel connected to their educational institution (Voelkl, 2012). It encompasses a sense of attachment, loyalty, and belongingness to the school community. It has been identified as a significant predictor of college adjustment and well-being.

Social Identity Theory (SIT) (Tajfel and Turner, 2004) posits that individuals derive a sense of identity and self-esteem from their membership in social groups, such as educational institutions. According to SIT, individuals strive to maintain a positive social identity by identifying with and positively valuing their group memberships. In this study, school identity can be seen as a social identity where students develop a positive emotional connection with their educational institution. SIT suggests that a strong school identity leads to positive outcomes, such as increased engagement, social integration, and well-being, contributing to college adjustment (Kelly, 2009). Students who identify strongly with their institution are more likely to participate in campus activities, form positive relationships with peers and faculty, and experience greater social support and connectedness (Kulp et al., 2021). This sense of belongingness and connectedness to the school community has been associated with higher levels of academic motivation, better academic performance, and increased overall well-being (Wormington et al., 2012).

**Figure 1:**
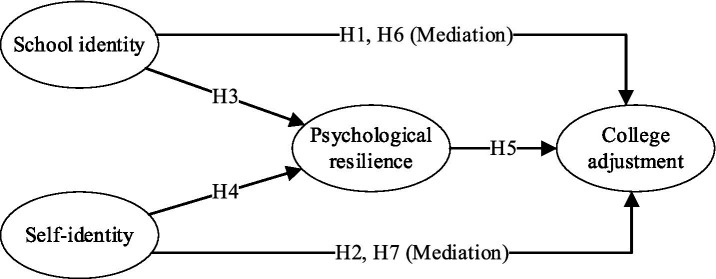
Hypothesized model.

School identity may be crucial in students’ adjustment to culture shock. During adversity, students who identify strongly with their institution may perceive themselves as part of a larger community providing support, resources, and stability (Drury, 2018). This sense of belongingness and attachment to the school can act as a protective factor, buffering the negative impact of emergencies on students’ adjustment and well-being (Figure 1). Therefore, this study hypothesizes that:

*H1*: School identity positively impacts college adjustment.

### Self-identity and college adjustment

Self-identity, often called personal identity or self-concept, encompasses an individual’s beliefs, values, interests, goals, and perceptions of oneself (Berzonsky, 1994). While cultural identity is a critical component for international students, the measure of self-identity used in this study captures a broader sense of personal coherence and continuity. It plays a fundamental role in shaping individuals’ behaviors, attitudes, and decision-making processes. In the college students’ adjustment context, self-identity has been recognized as a significant predictor of academic performance, psychological well-being, and overall adjustment (Xie et al., 2019).

Identity Consolidation Theory extends SIT by emphasizing the importance of identity development and consolidation in achieving positive adjustment outcomes. This theory posits that a coherent and integrated self-identity, encompassing personal values, beliefs, and goals, is crucial for individuals to navigate and adapt to various life domains (Schwartz, 2007), including the college environment. A well-defined self-identity gives individuals a sense of purpose, self-confidence, and resilience, enabling them to cope with challenges and promote positive adjustment effectively (Rajan-Rankin, 2014).

College students with a well-defined and positive self-identity tend to experience higher academic engagement, motivation, and satisfaction (Riswanto et al., 2022). Self-identity gives individuals a sense of purpose, direction, and meaning, which can enhance their commitment to academic goals and increase their resilience in the face of challenges (Rajan-Rankin, 2014). Moreover, individuals with a clear sense of self-identity are more likely to engage in adaptive coping strategies, seek support when needed, and display higher levels of self-efficacy (Robinson, 2021). These factors contribute to their overall adjustment and well-being in the college environment.

During culture shocks, self-identity can be critical in students’ coping and adaptation skills. Individuals with a well-defined self-identity may better understand their strengths, values, and personal resources, enabling them to navigate crises with resilience and adaptability (Son et al., 2020). They are more likely to maintain a positive outlook, proactively seek support, and engage in problem-solving strategies when faced with adversity. On the other hand, students with a weaker or less developed self-identity may struggle to cope with the challenges brought about by culture shock, experiencing heightened levels of distress and difficulties adjusting to the changing circumstances. Therefore, this study hypothesizes that:

*H2*: Self-identity positively impacts college adjustment.

### Mediating effect of psychological resilience

Psychological resilience refers to an individual’s ability to adapt and recover from adversity, challenges, and stressful situations (Masten, 2014). Importantly, this study conceptualizes resilience not as a fixed personality trait, but as a dynamic and developable process. This process-based perspective is crucial for its role as a mediator, as it suggests that resilience can be enhanced by antecedent factors like a strong identity. It encompasses personal characteristics, cognitive processes, and social support systems contributing to positive adaptation.

Students with a strong sense of school identity may experience a greater sense of belongingness, social support, and a connection to their educational institution (Kulp et al., 2021). These factors provide a protective buffer against stress and adversity, promoting the development of effective coping strategies and enhancing psychological resilience (Drury, 2018). Studies have shown that individuals with a well-defined self-identity have higher levels of psychological resilience (Rajan-Rankin, 2014). A clear understanding of one’s values, beliefs, and goals enables individuals to draw upon their strengths and resources during challenging times (Son et al., 2020). This self-identity serves as a foundation for building resilience and facilitates the ability to adapt and recover from adversity. Therefore, this study hypothesizes that:

*H3*: School identity positively impacts psychological resilience.

*H4*: Self-identity positively impacts psychological resilience.

Resilience theory suggests that individuals possess inherent abilities and resources to withstand and recover from adversity (Wang et al., 2015). As a construct within this theory, psychological resilience encompasses personal characteristics, cognitive processes, and social support systems that contribute to positive adaptation. According to resilience theory, individuals with higher levels of psychological resilience are better equipped to handle the demands of college life, navigate emergencies, and maintain their overall well-being, leading to improved college adjustment outcomes (Banyard and Cantor, 2004). Resilient individuals demonstrate positive adaptation, coping skills, and the ability to maintain their psychological well-being in the face of stressors (Brewer et al., 2019). Resilient students demonstrate higher levels of academic performance, engagement, and persistence, as well as better social integration and mental health outcomes (Ayala and Manzano, 2018). They are better equipped to handle the demands of college life, navigate emergencies, and maintain their overall well-being. Therefore, this study hypothesizes that:

*H5*: Psychological resilience positively impacts college adjustment.

Moreover, a strong school identity may promote the development of psychological resilience, which, in turn, facilitates successful adaptation to the college environment (Kelly, 2009). This pathway suggests that school identity indirectly impacts college adjustment through its influence on psychological resilience. A well-defined self-identity may enhance the development of psychological resilience, leading to improved college adjustment outcomes (Rajan-Rankin, 2014). This pathway suggests that self-identity indirectly impacts college adjustment through its influence on psychological resilience. Therefore, this study hypothesizes that:

*H6*: School identity positively impacts college adjustment through psychological resilience.

*H7*: Self-identity positively impacts college adjustment through psychological resilience.

## Methods

### Participants and procedures

Participants are non-Chinese international college students from Chinese colleges. Data were gathered through an online platform called “Questionnaire Star,” a reputable survey site in China, under the presumption of informed consent. 2,325 responses in total were gathered. The following three things were included as attention-screening questions, for example, “Please select four stars.” The data analysis used questionnaires with all three questions answered accurately. 2,097 questionnaires with a satisfactory response rate of 90.19% remained after questionnaires with invalid participants, and those that failed the attention test were eliminated. A post-hoc sensitivity analysis conducted with G*Power confirmed that the sample size 2,097 provides statistical power exceeding 0.99 to detect even small effect sizes (*f*^2^ ≥ 0.01), ensuring the robustness of our findings. There were 962 girls and 1,135 boys among them. Students could earn 5–10 Yuan through a random draw by participating. The review board approved all study procedures of the researchers’ institution (Table 1).

**Table 1:** Demographic information.

Demographic variable	Category	Frequency	Percentage (%)
Gender	Male	1,135	54.12
	Female	962	45.88
Age	<20 years	1,019	48.59
	21–25 years	1,040	49.60
	26–30 years	34	1.62
	>30 years	4	0.19
Grade	Undergraduate	1,942	92.61
	Postgraduate	155	7.39
College level	Vocational college	362	17.26
	General universities	1,460	69.62
	Double first-class universities	275	13.11
Total		2,097	100

### Measures

#### School identity

School identity was measured by the scale developed in the context of college students and verified in different studies (Ding, 2012). The scale contains eleven items, for example, “I think my school is an excellent university.” Likert-5 points scored for all the items. The higher scores show higher levels of school identification.

#### Self-identity

This study applied the self-identity scale developed initially by Ochse and Plug (1986). The scale includes six items. Likert-5 points scored for all the items. The higher scores show higher levels of self-identification. One example item is “I feel that my lifestyle suits me well.”

#### Psychological resilience

To gauge the participants’ resilience, we employed the Brief Resilience Scale. It assesses resilience as the ability to recover or bounce back from stressful or difficult situations (Smith et al., 2008). Participants were prompted to select their responses depending on how well their responses described their current circumstances in the face of challenges and setbacks. The statement “I think adversity has a motivating effect” exemplifies a resilient description. The scale comprises twelve items. Likert-5 points scored for all the items. The excellent results show high levels of resilience.

#### College adjustment

College adjustment was evaluated by the General Adaptation Scale for international students (Polat and Arslan, 2022), which considered cultural adjustment as part of college adjustment for international students. Four dimensions of college adjustment were measured in this research. The students were prompted to select a response and indicate how much they agreed or disagreed with each statement. “I feel like I am getting more and more comfortable with college studies” is an example. Likert-5 points scored for all the items. Higher scores indicate a better adjustment to college.

### Data analysis

Data analysis commenced with preliminary steps to ensure measurement integrity. Confirmatory factor analyses (CFA) were conducted using Mplus 8.0 to assess the structural validity of the measures. Internal consistency reliability was subsequently examined by calculating Cronbach’s alpha coefficients with SPSS 26.0. Following these assessments, descriptive statistics and intercorrelations among all variables were computed using SPSS 26.0. To test the main effects hypothesized in H1–H5, structural equation modeling (SEM) was performed using Mplus 8.0. Based on the preliminary correlation analysis (Table 2), which showed that gender was correlated with psychological resilience and college-level was correlated with college adjustment, both variables were included as control variables in the main structural equation model to ensure the robustness of our findings and rule out their potential confounding effects. For the estimation of indirect coefficients, a bootstrapping procedure with 5,000 replications was employed (Preacher and Hayes, 2004). This method allowed for calculating mediating effect sizes and their 95% bias-corrected confidence intervals, with significance determined if the confidence interval did not include zero (Preacher and Hayes, 2004).

**Table 2:** Correlations among variables (*N* = 2,097).

Variable	1	2	3	4	5	6	7	8
1. Gender								
2. Age								
3. Grade								
4. College level								
5. School identity	0.026	−0.026	0.009	−0.029	1			
6. Self-identity	0.004	−0.030	0.009	−0.044*	0.805**	1		
7. Psychological resilience	0.045*	−0.021	0.001	−0.039	0.636**	0.657**	1	1
8. College adjustment	−0.016	−0.026	0.015	−0.088**	0.803**	0.862**	0.716**	1
Mean	1.459	20.856	1.706	1.959	3.534	3.487	3.713	3.482
Standard deviation	0.498	1.871	0.596	0.550	0.765	0.741	0.780	0.729

(1) Gender: female = 1, male = 2; Grade: 1 = Undergraduate, 2 = Post graduate; College level: 1 = Vocational college, 2 = General universities, 3 = Double first-class universities. (2) *p < 0.05, **p < 0.01, two-tailed.

## Results

### Validity and reliability test

The structural validity of the measurements was rigorously examined through a series of confirmatory factor analyses. To enhance the stability of latent estimates, two or three indicators were randomly generated for each latent construct (Xia et al., 2022). Table 3 presents the results (χ^2^ = 7817.088, χ^2^/df = 13.294; RMSEA = 0.077, 90% CI = (0.075, 0.078); CFI = 0.903; TLI = 0.896; SRMR = 0.045), indicating that the hypothesized four-factor model exhibited a superior fit to the data compared to three competing models, thus establishing strong structural validity. It is important to note that the CFI and TLI values for the competing one-, two-, and three-factor models (Models 2–4) were below the conventional threshold of 0.90. This outcome is expected, as these models represent theoretically less plausible structures where distinct constructs are forcibly combined. The poor fit of these alternative models, in contrast to the superior fit of our hypothesized four-factor model, provides strong evidence for the discriminant validity of our four core constructs. However, the χ^2^/*df* value did not fit the threshold of under 3; the hypothesized four-factor model showed the smallest value among the four models. It is important to note that the χ^2^/*df* value is heavily impacted by sample size, with a larger sample size resulting in a higher χ^2^/*df* value (Segars and Grover, 1993); the sample size in this research was 2,097. The measures showed good structure validity, as the hypothesized four-factor model fit the data better than the other three models.

**Table 3:** Results of validity testing.

Model	Factor	χ^2^ χ^2^/*df*	△χ^2^	RMSEA 90% CI	CFI	TLI	SRMR
1	School identity, Self-identity, Psychological resilience, College adjustment	7,817.008 13.294		0.077 0.075, 0.078	0.903	0.896	0.045
2	School identity, Self-identity, Psychological resilience + College adjustment	14,477.62 24.497	6660.612**	0.106 0.104, 0.107	0.813	0.801	0.072
3	School identity + Self-identity, Psychological resilience + College adjustment	16,357.394 27.584	8540.386**	0.113 0.111, 0.114	0.788	0.775	0.074
4	School identity + Self-identity + Psychological resilience + College adjustment	19,915.548 33.528	12,098.54**	0.125 0.123, 0.126	0.740	0.725	0.082

Concurrently, the internal consistency reliability of the measures was assessed using Cronbach’s alpha coefficients; all values were found to be adequate, with thresholds met at 0.70 (Taber, 2018). The results showed Cronbach’s alpha values were 0.948 for school identity, 0.904 for self-identity, 0.945 for psychological resilience, and 0.971 for college adjustment.

Furthermore, to ensure the stability of our proposed model, a robustness check was conducted using a random split-half method (Johnson and Christensen, 2024). The total sample (*N* = 2,097) was randomly divided into two subsamples (Subsample A, *n* = 1,049; Subsample B, *n* = 1,048). The hypothesized four-factor model was then tested on each subsample separately. The results from both subsamples demonstrated a good fit and were highly consistent with the results from the full sample (Subsample A: CFI = 0.905, TLI = 0.898, RMSEA = 0.076; Subsample B: CFI = 0.901, TLI = 0.894, RMSEA = 0.078). All key path coefficients remained significant and in the same direction across both subsamples. This consistency confirms the robustness of our model structure.

### Correlation and descriptive information

Table 2 shows the correlations and descriptive statistics for all variables. Both school identity and self-identity were positively correlated with psychological resilience (school identity: *r* = 0.636, *p* < 0.01; self-identity: *r* = 0.657, *p* < 0.01). Psychological resilience was positively correlated with college adjustment (*r* = 0.716, *p* < 0.01). For control variables, college-level was correlated with college adjustment, and gender was correlated with psychological resilience; thus, college-level and gender were controlled in the associated hypothesis test.

### Hypothesis test

**Figure 2:**
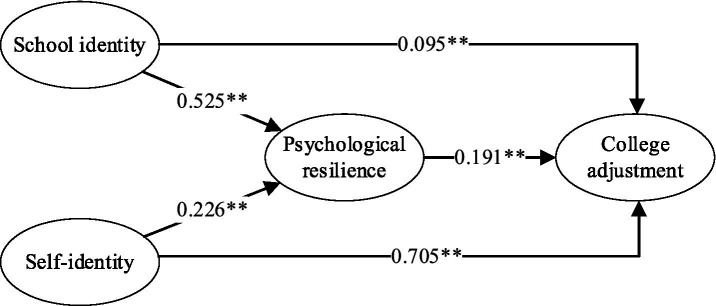
Results of the main effect.

As illustrated in Figure 2, the direct effects largely supported our hypotheses. Specifically, H1 and H3 were supported by a significant positive association between school identity and psychological resilience (*β* = 0.525, *p* < 0.01). Consistent with H2 and H4, self-identity also exhibited a significant positive relationship with psychological resilience (*β* = 0.226, *p* < 0.01). Additionally, H5 received support, indicating that psychological resilience significantly and positively predicted college adjustment (*β* = 0.191, *p* < 0.01).

Bootstrapping analyses were performed to examine the proposed mediating role of psychological resilience in the relationships between school identity, self-identity, and college adjustment. As detailed in Table 4, the results supported the mediating effects hypothesized in H6 and H7, as their 95% bias-corrected confidence intervals did not include zero. The final mediation model accounted for a significant portion of the variance in the outcome variable. Specifically, the model explained 78.5% of the variance in college adjustment (*R*^2^ = 0.785), indicating a substantial overall effect.

**Table 4:** Results of mediating effects.

Path	Effect	Standard error	95% LLCI	95% ULCI
School identity → Psychological resilience → College adjustment	0.100	0.017	0.074	0.130
Self-identity → Psychological resilience → College adjustment	0.043	0.013	0.025	0.067

## Discussion

This study aimed to unravel the intricate relationships between identity (school and self), psychological resilience, and college adjustment among international students navigating the unique challenges of culture shock in Chinese universities. Our findings provide robust empirical support for the hypothesized model, shedding light on the crucial mechanisms through which a strong sense of self and cultural belonging contribute to successful adaptation in a cross-cultural educational environment.

### Identity and psychological resilience in the face of culture shock

The findings robustly confirm Hypotheses 1 and 3, demonstrating that international students’ strong identification with their host university serves as a significant positive predictor for both their psychological resilience and overall college adjustment. For these students, a deep sense of school identity cultivates a crucial feeling of belongingness, attachment, and loyalty to their new academic home (Verhoeven et al., 2019). This strong identification is a vital protective factor, particularly during unforeseen challenges or emergencies, enabling students to draw strength from the perceived availability of institutional support, shared resources, and a collective sense of stability (Luehmann, 2009). Consequently, international students with a more substantial university identity tend to exhibit heightened psychological resilience, enhancing their ability to navigate demanding circumstances and adapt to changes. Moreover, this robust connection to the institution translates into better academic engagement, more successful social integration into the host culture, and improved overall well-being, even when confronting adverse situations (Brown and Shay, 2021). However, it is worth considering potential boundary conditions for this positive effect. For instance, in an institutional environment that is unsupportive or fails to meet students’ expectations, a strong school identity could potentially become a source of disillusionment or stress, thereby negatively affecting adjustment. Acknowledging such complexities suggests that the relationship between school identity and well-being is not universally positive and depends on the quality of the student-institution relationship.

Similarly, Hypotheses 2 and 4, proposing that self-identity positively influences psychological resilience and college adjustment, were also strongly supported. Self-identity, encompassing an individual’s personal values, beliefs, and goals, is crucial for international students who often experience a re-evaluation of their self-concept during cross-cultural adaptation (Robinson, 2021). Aligned with Identity Consolidation Theory (ICT), a well-defined and coherent self-identity provides a sense of purpose, self-confidence, and internal consistency. This clarity about who they are and what they value equips students with the inner resources to face the unexpected challenges and emotional fluctuations of culture shock. They are more likely to engage in proactive coping strategies, maintain a positive outlook, and derive meaning from difficult experiences, which directly enhances their psychological resilience (Rajan-Rankin, 2014). This enhanced resilience, in turn, allows them to sustain academic engagement, build meaningful social connections, and manage personal-emotional well-being more effectively within the Chinese university context.

### The role of psychological resilience in college adjustment

The findings indicate that psychological resilience significantly and positively predicts college adjustment (Hypothesis 5). This result is particularly significant for international students, as culture shock inherently presents a high degree of adversity and uncertainty. Students with higher levels of psychological resilience are better equipped to perceive cultural misunderstandings, academic pressures, and social isolation not as insurmountable obstacles but as manageable challenges (Azpeitia et al., 2025). Their capacity for adaptive coping, optimistic thinking, and emotional regulation enables them to “bounce back” from setbacks, learn from their experiences, and maintain their psychological well-being. This adaptive capacity directly translates into better academic performance, smoother social integration, and healthier emotional states, all contributing to a more successful overall college adjustment experience in China (Gao et al., 2024).

While our model emphasizes the role of internal psychological resources, it is crucial to acknowledge that these resources do not operate in a vacuum. External socioeconomic factors, particularly financial stability, can significantly impact a student’s capacity for resilience. For the large proportion of self-financed international students in China (approximately 87% in 2018), the constant pressure of tuition fees and living expenses can act as a chronic stressor, potentially depleting the cognitive and emotional reserves needed to cope with culture shock. In contrast, students on full scholarships may have a greater capacity to focus on their academic and social adjustment. This suggests that for resilience-building interventions to be effective, they must be complemented by university support systems that address students’ practical and financial challenges, thereby creating a more equitable foundation for well-being.

Crucially, our study confirms the mediating role of psychological resilience, as Hypotheses 6 and 7 suggested. Our results suggest that the roles of school identity and self-identity in college adjustment are not merely direct; instead, these forms of identity also appear to operate indirectly through their significant positive association with psychological resilience. This implies a powerful indirect pathway: a strong cultural identity provides a stable foundation and a sense of belonging. At the same time, a clear self-identity offers internal coherence and purpose. Both forms of identity strengthen an international student’s inherent capacity to cope with the specific stressors of culture shock, thereby building their psychological resilience. This strengthened resilience directly facilitates their comprehensive adjustment to the demands of living and studying in a Chinese university. This finding underscores that fostering identity development is not an end in itself for adaptation, but a critical precursor to building the resilient mindset necessary to thrive in a challenging new environment (Haktanir et al., 2021).

Furthermore, it is important to consider the cultural context in which these psychological processes unfold. The relative importance of self-identity versus school identity in fostering resilience may differ for students from individualistic versus collectivistic cultural backgrounds (Zhou et al., 2025). For instance, students from collectivistic societies might derive a greater sense of resilience from their connection to a group (i.e., school identity). In contrast, those from individualistic cultures may rely more on a consolidated sense of personal self (i.e., self-identity). Although our study did not analyze these potential variations, this remains a crucial avenue for future cross-cultural research, which could leverage our existing dataset on students’ nationalities.

### Theoretical contributions

This study offers several significant theoretical contributions to the fields of cross-cultural psychology, student development, and international education research.

Firstly, the most significant theoretical contribution lies in empirically demonstrating the mediating role of psychological resilience in the relationship between identity and college adjustment among international students facing culture shock in China. While previous research has separately acknowledged the importance of identity and resilience for adaptation, the direct causal pathway and the precise mechanism by which a strong sense of identity facilitates adjustment via enhanced resilience have remained mainly underexplored. This study elucidates a crucial psychological process: a well-defined and stable identity is a foundational resource, fostering an individual’s capacity to “bounce back” from adversity, directly translating into more successful adaptation to the new academic and social environment. This moves beyond correlational understandings, providing a more nuanced and mechanistic explanation of cross-cultural adjustment.

Secondly, this research extends and applies established theories, namely Social Identity Theory and Identity Consolidation Theory, to a specific and under-researched population: international students in the unique context of China. Our finding that a strong school identity—a form of positive group affiliation—is a significant predictor of both resilience and adjustment provides robust empirical support for SIT’s proposition that individuals derive psychological resources from their social group memberships (Tajfel and Turner, 2004). Similarly, the crucial role of a coherent self-identity in fostering resilience aligns directly with Identity Consolidation Theory’s emphasis on a consolidated sense of self as a prerequisite for navigating significant life transitions effectively (Schwartz, 2007). By showing that a coherent sense of self (identity consolidation) and positive group affiliations (social identity) predict resilience in a cross-cultural setting, the study validates the enduring relevance of these theories in understanding dynamic identity processes during periods of significant life transition and cultural immersion. It demonstrates how the negotiation of multiple identities (e.g., home culture vs. host culture) can, when consolidated effectively, act as a psychological buffer against the stressors of culture shock, rather than solely being a source of conflict.

Thirdly, this study contributes to the growing literature on non-Western international education contexts by focusing on international students in China. Most theories of cross-cultural adaptation have historically been developed and tested in Western settings. Investigating these constructs within China’s distinct cultural, social, and educational landscape offers valuable insights into the universality and cultural specificity of identity development, resilience, and adjustment processes. The large sample size from diverse universities across China further strengthens the generalizability of these findings within this particular context, providing a robust empirical foundation for future cross-cultural comparative studies.

Finally, this study enriches the understanding of college adjustment models by integrating psychological constructs (identity, resilience) often studied in isolation. It highlights that successful adjustment is not merely a function of external support or exposure. However, it is profoundly influenced by internal psychological resources and how they are leveraged. This integrated model provides a more holistic framework for understanding student success beyond academic performance, encompassing psychological well-being and social integration.

### Practical implications

The findings of this study offer direct and actionable implications for various stakeholders involved in supporting international students in China, including universities, student support services, and policymakers.

Firstly, given the identified mediating role of psychological resilience, universities and student support services should prioritize developing resilience-building programs for international students. These programs could include workshops on stress management, emotional regulation, problem-solving skills, mindfulness, and adaptive coping strategies. Integrating these into pre-arrival orientations or early-semester curricula could equip students with essential tools before the full impact of culture shock.

Secondly, the strong link between identity and resilience suggests the importance of fostering a strong and integrated sense of identity among international students. Universities can facilitate this by: (1) Creating spaces for cultural sharing and exchange: Encouraging students to share their home cultures while engaging with Chinese culture can help them negotiate and consolidate their bicultural identity. (2) Developing mentorship programs: Pairing new international students with senior international students or local Chinese students can provide social support, reduce feelings of isolation, and offer guidance on navigating cultural nuances, thereby strengthening their social identity within the university community. (3) Promoting self-reflection and personal growth: Workshops or counseling services that encourage students to explore their values, strengths, and goals in the context of their new environment can contribute to identity consolidation. Specific interventions could include guided journaling exercises, narrative therapy workshops where students articulate and reframe their cross-cultural journeys, or strengths-based coaching to help them align their academic and personal goals with their core identity. Such identity-affirming practices serve as a solid foundation for resilience-building.

Thirdly, the research highlights the necessity of holistic support systems that address adjustment’s psychological and social aspects. Beyond academic support, universities should ensure readily accessible and culturally competent mental health services. Counselors should be trained to understand international students’ unique challenges, including issues related to identity negotiation, culture shock, and potential feelings of alienation or homesickness.

Fourthly, for policymakers and university administrators in China, these findings underscore the importance of investing in a comprehensive international student support infrastructure. This includes recruitment efforts and sustained investment in student well-being from admission through graduation. Tailored interventions based on these findings can significantly reduce attrition rates, improve student satisfaction, and enhance China’s reputation as a welcoming and supportive destination for global talent.

Finally, the study provides valuable insights for international students and their families. Understanding that a strong sense of identity and developing psychological resilience are key to successful adjustment can empower students to proactively engage in self-reflection and seek resources that foster these qualities, enabling a more positive and enriching experience in China.

## Conclusion

This study investigated the complex interplay between identity, culture shock, psychological resilience, and college adjustment among international students in China. Our findings robustly confirm the hypothesized model, demonstrating that psychological resilience significantly mediates the relationship between a strong sense of identity and successful college adjustment, even in the presence of culture shock. Specifically, a coherent and consolidated identity was found to predict higher levels of psychological resilience positively. Higher resilience, in turn, was directly associated with better overall adjustment to the university environment in China, encompassing academic, social, and personal-emotional adaptation. Crucially, this resilient pathway appears to buffer the negative impacts of culture shock, suggesting that internal resources are vital for navigating cross-cultural challenges.

These findings significantly advance our theoretical understanding of cross-cultural adaptation by empirically demonstrating the mediating role of psychological resilience. This research extends established theories such as Social Identity Theory and Identity Consolidation Theory by applying them to the unique and under-researched context of international students in China, illustrating how a strong sense of self and positive group affiliations can enhance adaptive capacities. It provides a more integrated model of college adjustment, highlighting that successful integration is not merely a function of external support but is profoundly influenced by the effective leveraging of internal psychological resources. The practical implications of this research are substantial for universities, student support services, and policymakers in China dedicated to supporting their growing international student population. The findings underscore the critical need for proactive interventions fostering identity exploration and resilience-building among international students. Programs focused on cultural identity consolidation, cross-cultural communication skills, stress management, emotional regulation, and problem-solving should be prioritized to equip students with the necessary psychological tools. Furthermore, universities should cultivate environments encouraging cultural exchange and providing culturally competent mental health services, ensuring a holistic support framework that transcends academic assistance to encompass psychological well-being and social integration.

Despite its significant contributions, this study is subject to certain limitations. While our mediation model provides strong support for the proposed relationships, the cross-sectional design limits our ability to infer definitive causality; longitudinal studies are needed to confirm the dynamic pathways over time. Reliance on self-report measures may introduce common method bias and social desirability effects. Additionally, although a large sample was drawn from diverse universities, the findings are specific to the international student experience in China. Without further research, they may not be directly generalizable to other host countries or student populations. Future research should employ longitudinal designs to more definitively establish the causal relationships and track the development of identity and resilience throughout international students’ academic journeys. Qualitative methodologies could offer richer insights into the lived experiences of international students, exploring how identity is negotiated and resilience is cultivated in the face of specific cultural challenges unique to the Chinese context. Intervention studies testing the efficacy of specific identity- or resilience-building programs would provide direct evidence for their practical utility. Thirdly, a limitation of the current study is that while data on the participants’ country of origin were collected, an analysis of how cultural background (e.g., individualism vs. collectivism) might moderate the proposed relationships was not performed, as it was beyond the scope of our primary research questions. This means that potential cultural nuances in the interplay between identity, resilience, and adjustment are not captured in our model. Future research, including secondary analyses of the current dataset, should explore these important cultural variations to provide a more fine-grained understanding of the cross-cultural adjustment process. Finally, comparative studies across different host countries or with diverse international student demographics could further illuminate cultural specificities and universal principles governing cross-cultural adjustment.

In conclusion, this research underscores the profound importance of investing in the psychological well-being of international students. By understanding and nurturing their internal psychological resources, particularly their sense of identity and psychological resilience, universities in China can foster a more supportive, enriching, and successful educational journey for this increasingly vital demographic, transforming potential challenges into opportunities for growth and successful global integration.

## Data Availability

The raw data supporting the conclusions of this article will be made available by the authors, without undue reservation.
